# The convenient method and application for monitoring the health of traffic sign poles based on mobile phone

**DOI:** 10.1038/s41598-023-47400-5

**Published:** 2023-11-23

**Authors:** Xiao Xie, Yumin Chen, Changchun Li, Junwei Guo, Saeed Sarajpoor

**Affiliations:** 1 College of Civil and Transportation Engineering, Hohai University, Nanjing, 210098 China; 2 Institute for Smart City of Chongqing University in Liyang, Liyang, 213300 China

**Keywords:** Mechanical engineering, Engineering, Mathematics and computing, Physics

## Abstract

Traffic sign poles are crucial components of the highway system, and their maintenance mainly relies on the subjective judgment of staff, which is low-efficiency and will lead to mistaken inspections. This paper proposes a convenient and effective method to monitor the health of sign poles by using a mobile phone. It is unknown whether a mobile phone can be used as a conventional acceleration sensor. Therefore, the performance of the mobile phone was initially tested to ensure its suitability for acquiring the acceleration data of the traffic sign pole. The results show that the acceleration sensor of mobile phones is high-performance and can be used as a traditional acceleration sensor under the similar sampling frequency. The mobile phone can measure the 1st, 2nd and 5th natural frequencies of the traffic sign pole. Although the 1st and 5th natural frequencies have a large error with simulation, the 2nd natural frequency is accurate and stable. The looseness of the base connection greatly impacts on the 2nd natural frequency, which can be used as a measure of the loose base connection. The 2nd natural frequency was measured for 21 times and found to conform to a normal distribution. The relationship between the 2nd natural frequency and base connection loosening was investigated, which fitted to the equation. The fitting result is good and can be used to predict the degree of sign pole base connection loosening. Therefore, the mobile phone based convenient health monitoring method for traffic sign poles is feasible.

## Introduction

As an important part of the highway system, traffic sign poles play the role of indication, warning and prohibition^1–5^. According to statistics^6,7^ , the total mileage of roads in China is 5 million kilometers, of which 77.6% is built before 2011. Traffic signal support structures consistently undergo wind-induced excitation, and the subsequent vibrations result in stress reversals can lead to fatigue and fracture, particularly in the welded connections^8^ . Therefore, the normal life expectancy of a traffic sign pole is ten years, meaning that many sign poles will need to be maintained or replaced^9^ . Currently, the maintenance and repair of traffic sign poles rely mainly on the subjective judgment of staff, which can lead to excessive randomness and can easily result in missed or mistaken inspections. Natural frequency refers to the natural vibration frequency of the elastomer or elastic system itself, which is related to its own mass and stiffness, boundary support conditions and vibration form^10–14^ . When the structure is aged or the base connection is loose, its natural frequency will change^15–18^. There is a certain relationship between the change in natural frequency and the degree of structural deterioration, so the natural frequency is often used as an indicator to measure the health level of the structure. Hamilton III^19^ determined the dynamic characteristics of a traffic signal structure by accelerometers and self-frequencies, and found that adding damping devices is able to reduce the structural fatigue caused by wind-induced oscillations. McManus et al^20^ further explored dynamic characteristics of a cantilevered traffic signal structure under forced vibration experimentally and theoretically, and it was clear that computer modeling can facilitate an understanding of the application of static and dynamic loading to different geometric configurations. Bao et al^21^ studied the scour damage of bridges by the change in natural frequency of bridge piers. Zhang et al^22^ identified cracks in cantilever beams by studying the variation of natural frequency. ORAI et al^23^ realized a simple method to monitor the health of power poles by the acceleration sensor of the iPod. Therefore, it is possible to use the natural frequency as the health evaluation criterion of traffic sign poles^24–28^ . At present, obtaining the natural frequency of structures usually requires professional equipment such as computer, acceleration sensors, dynamic signal testing and analysis system. The testing process is time-consuming and is difficult to operate outside. Therefore, the traditional method is not suitable for the rapid inspection of traffic sign poles. It means that it is extremely meaningful to develop a simple and fast method to obtain the natural frequency of traffic sign poles. The innovation of this paper is to use the mobile phone as a device to obtain the natural frequency of the target structure, which makes fast and accurate measurements possible. Mobile phone is the most common device with a three-way acceleration sensor^29,30^ , and is equipped with an acceleration sensor and LAN function, which can realize the linkage of multiple devices within a certain range, and can save or upload data. However, the acceleration sensor is designed to measure the acceleration of the phone itself. It is uncertain whether the mobile phone can measure the acceleration of other objects, and no software can directly obtain the acceleration data from a mobile phone. Therefore, this paper develops a software that can obtain the acceleration data of mobile phones. The mobile phones were compared to a conventional accelerometer sensor by shaking table test. And it was found that although the peak value of the mobile phone accelerometer data was slightly smaller than the actual value, the waveform was highly consistent with the actual and could reflect the frequency of the vibration. This test verifies the reliability of mobile phones as acceleration sensors. The test process is as follows. First, determine the sampling frequency of the mobile phone acceleration sensor. Then, in order to obtain the natural frequencies of different orders of the structure, the vibration signals of traffic sign poles are collected by using different sampling frequencies ac-cording to the Nyquist sampling theorem. The Nyquist sampling theorem explains the relationship between sampling frequency and signal spectrum. To ensure that the sampled digital signal retains the information in the original signal completely, in practical applications, the sampling frequency is ensured to be twice the highest frequency of the signal^31^ . The simulation model is established for modal analysis and compared with the measured value. After that, the relationship between the degree of structural deterioration and the change of natural frequency is obtained, and proposed a method for predicting. Finally, a convenient method for the health detection of traffic sign poles is developed.

## Development and verification of vibration monitoring software

### Information of mobile phone

**Figure 1:**
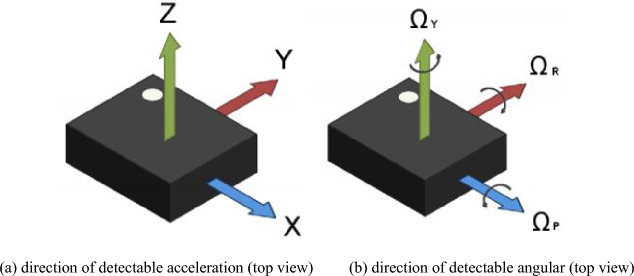
Measuring direction of the sensor.

The mobile phone selected in this paper is Xiaomi 11, HONOR9 and HUAWEI nova3, which are equipped with LSM6DSO, LSM6DS3 and LSM6DS3-C acceleration sensors. The sensors are a system-in-package featuring a 3D digital accelerometer and a 3D digital gyroscope. They support main OS requirements, offering real, virtual and batch sensors with 9 Kbytes for dynamic data batching. ST’s family of MEMS sensor modules leverages the robust and mature manufacturing processes already used to produce micromachined accelerometers and gyroscopes. It is finely calibrated to align with the characteristics of the sensing element^32^. The specific parameters are shown in Table 1 and the directions are defined in Figure 1.

**Table 1:** Performance parameters of mobile phone acceleration sensors.

Phones	Accelerometer	Acceleration range (g)	Gyroscope range (dps)	Sampling frequency (Hz)	Supply voltage (V)	Current consumption (mA)	Size ($mm^3$)
Xiaomi 11	LSM6DSO^32^			417		0.55	
HONOR 9	LSM6DS3	±16	±2000	250	1.71-3.6	0.90	2.5$\times$3$\times$0.83
HUAWEI nova 9	LSM6DS3-C^33^			250		0.90	

### Software development

**Figure 2:**
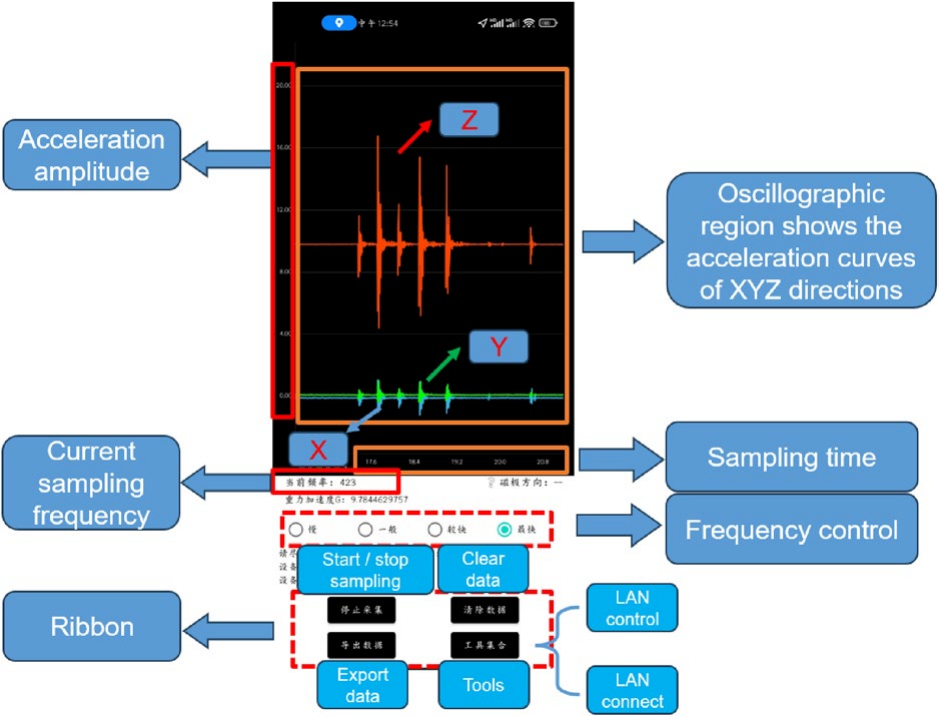
Interface of the acceleration detection software.

**Figure 3:**
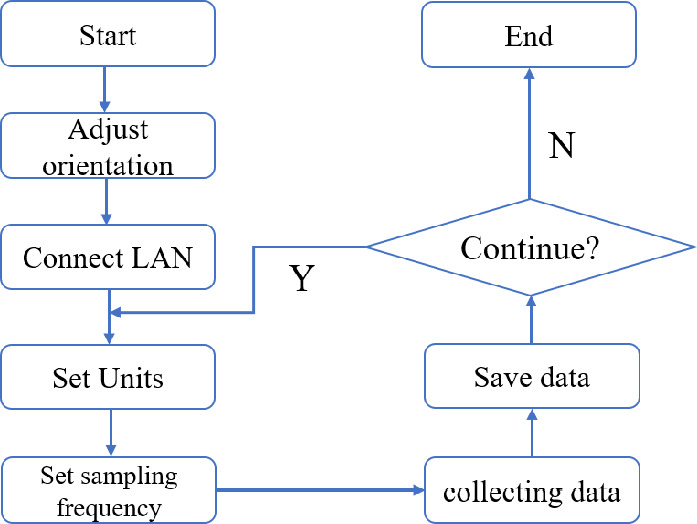
Software flowchart.

This paper uses self-developed acceleration detection software for data logging, which supports Android 9 or above. The software is able to read the data from the acceleration sensor of the mobile phone and record in real time. The sampling frequency of the mobile phone is divided into four levels: “Slow” is 5Hz; “General” is 15 Hz; “Fast” is 50 Hz; The “fastest” is 417 Hz for Xiaomi 11 and 250Hz for HONOR 9 and HUAWEI. The software can also provide the gravitational acceleration of the current position and the tilt angle of the phone, as shown in Figure 2. The software can not only obtain the acceleration in the XYZ direction of the mobile phone, but also decompose the acceleration into four directions, east, west, north and south, through GPS and mobile phone location system. In addition, the software also supports the control and linkage between devices under the same LAN. That is, within the LAN, one device can control the sampling, data storage and data upload of multiple devices. This function can effectively avoid the impact of the manual operation on data, and enables the linkage of multiple devices in a large area. The usage process is as follows, firstly, open the software and adjust the direction of the phone based on the measured object, then select the acceleration unit (g or $m/s^2$), and the sampling frequency to start collecting data. If the LAN function is utilized, all functions can be used at the controller. The specific operation process is shown in Figure 3.

### Performance test

**Figure 4:**
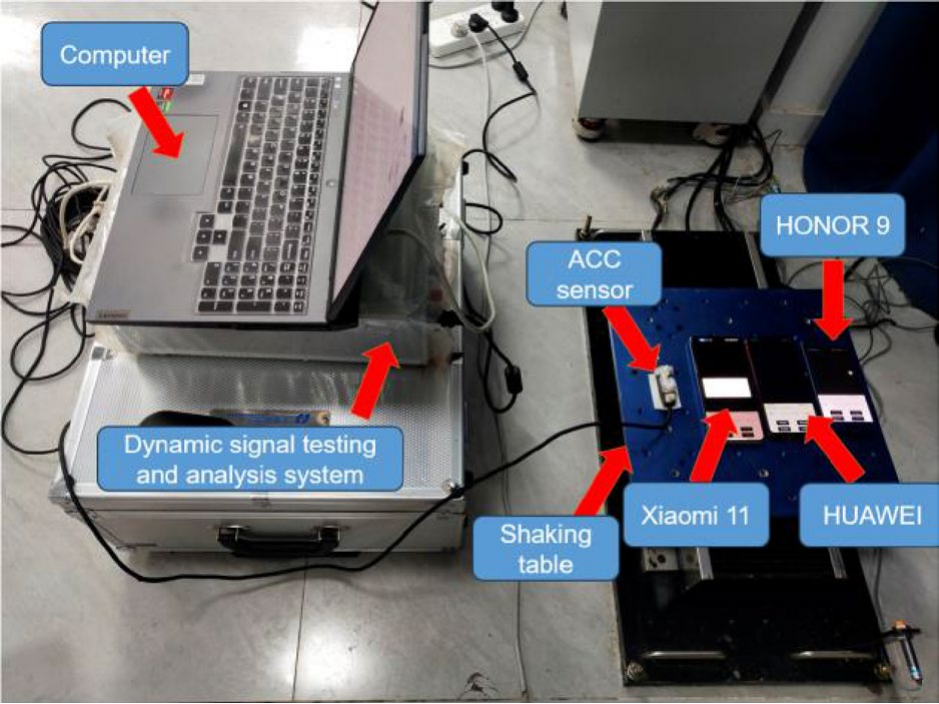
Shaking table test.

**Figure 5:**
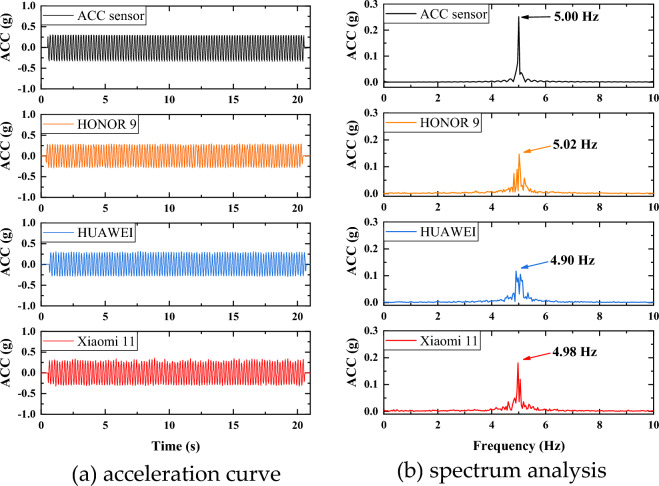
Comparison of mobile phones and acc sensor.

As mobile phones are not professional acceleration measurement tools, it is uncertain whether they can be used as acceleration sensors. This paper tests the acceleration measurement capability of the mobile phone by shaking table test. First, fix the acceleration sensor and mobile phone on the shaking table, as shown in Figure 4. The traditional acceleration sensor used in this paper supports a sampling frequency of up to 256kHz, with an error rate of less than 0.5%. In order to match the performance of the mobile phone acceleration sensor, only 500Hz was set in the experiment. Then start the shaking table and operate it at a frequency of 5Hz and with an acceleration of 0.5g. The mobile phone and acceleration sensor record data at 417Hz (Xiaomi 11), 250Hz (HONOR 9 and HUAWEI) and 500Hz, respectively, as shown in Figure 5. It can be seen from the data comparison between the mobile phones and the acceleration sensor that although the peak acceleration recorded by the mobile phones is slightly smaller than the acceleration sensor, their waveforms are highly consistent. Overall, Xiaomi 11 has a higher sampling frequency, so its frequency and acceleration amplitude are closer to the acceleration sensor, while the frequency of HONOR 9 is also close to 5Hz, but the acceleration amplitude is small. The frequency deviation of HUAWEI is larger and peak is not significant. The Pearson correlation coefficient is introduced to describe the degree of correlation between the two curves. The Pearson correlation coefficient captures the degree of linear correlation between two variables and has a value between -1 and 1. A negative number represents a negative correlation, and a positive number indicates a positive correlation, and a higher absolute value indicates a stronger correlation^34–39^. According to the calculation, the Pearson correlation coefficient of curves between Xiaomi 11 and acceleration sensor is 0.81, which shows a very strong correlation. Therefore, it can be concluded that the Xiaomi 11 can be used as an accelerometer sensor under a similar sampling frequency.

## Field test and result analysis

### Field test

**Figure 6:**
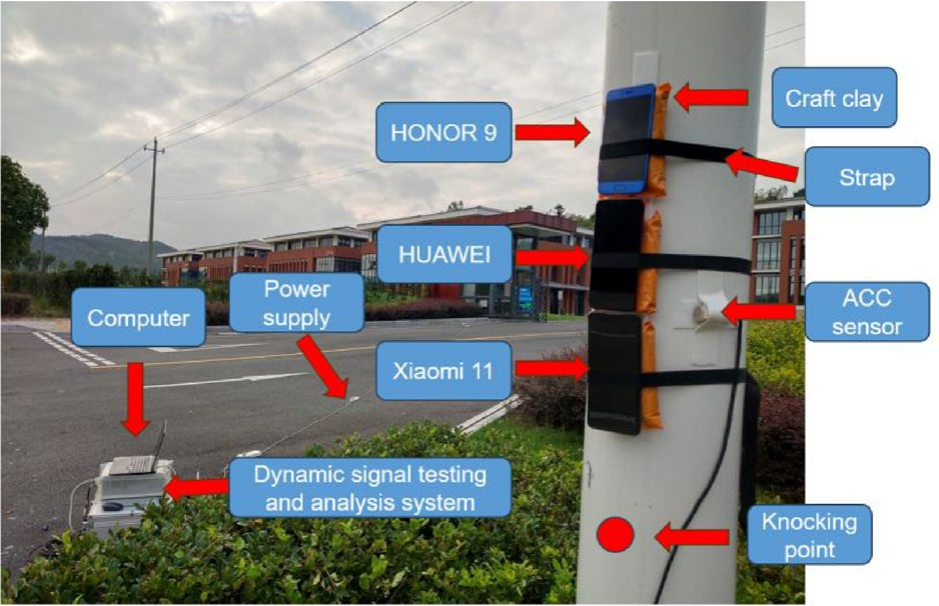
Site layout.

The test object selected in this paper is the cantilever beam traffic sign pole that has been built for one year, and the specific dimensions are shown in Figure 8. The test steps are as follows: First, fix the mobile phones 1.5m above the ground. Craft clay was placed between the mobile phone and the sign pole to ensure a tight fit. To avoid manual data manipulation, the LAN function was used to control the on and off of the mobile phone sampling. Then knock with a rubber hammer at 0.2m directly below the mobile phone. Finally, in order to obtain different natural frequencies of traffic sign pole, the above steps are repeated by changing the sampling frequency according to the Nyquist sampling theorem. The site layout is shown in Figure 6.

### Result analysis

**Figure 7:**
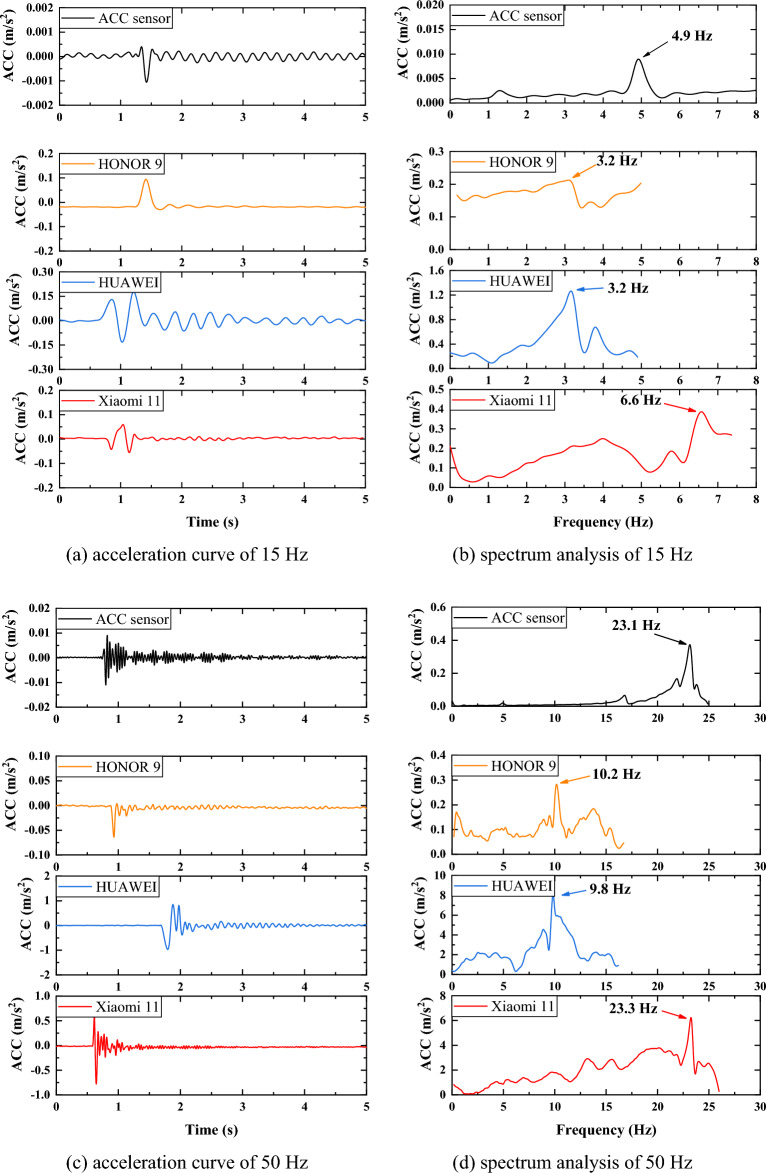

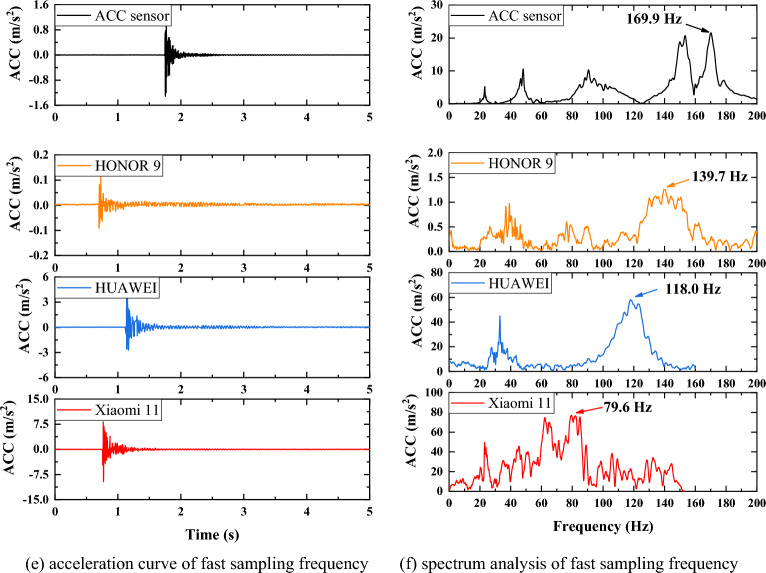
Comparison of mobile phones and acc sensor.

Since the rubber hammer is striking in the direction parallel to the Z direction of the mobile phone in the field test, the acceleration in this direction is collected. According to the Nyquist sampling theorem and performance of the mobile phones, sampling frequencies are taken at 15Hz, 50Hz and 417Hz (Xiaomi11), 250Hz (HONOR 9 and HUAWEI), respectively. Acceleration curves and spectrum analysis are shown in Figure 7. It can be seen that at a sampling frequency of 15Hz, the self- frequencies of HONOR 9 and HUAWEI are both 3.2Hz, while Xiaomi 11 is 6.6Hz, but the frequency of all of them differs from that of the acceleration sensor by ±1.7Hz. When the sampling frequency is 50Hz, the self-frequencies obtained from the Xiaomi 11 and the accelerometer are almost identical, 23.3Hz and 23.1Hz, respectively. The self-frequencies obtained by HONOR 9 and HUAWEI are also basically the same, but they are significantly smaller, at only about 10Hz. When using the highest sampling frequency, there is an obvious difference in the self-frequencies of the three mobile phones. The reason for the significant difference may be that the sampling frequency will affect the accuracy of the acceleration sensor of phones, resulting in inaccurate spectral analysis.

## Health analysis of traffic sign poles

### Modal analysis

**Figure 8:**
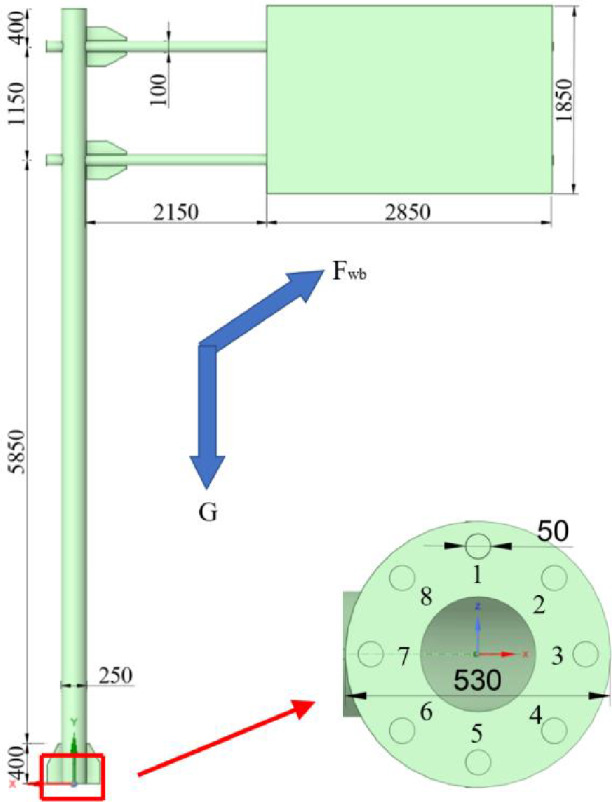
Site layout.

ANSYS Workbench is a universal finite element software for solving various practical simulation problems, mainly including structural static analysis, dynamic analysis, nonlinear analysis. ANSYS is suitable for building complex models. It can simulate the real situation by adjusting model parameters or applying loads^40–44^. Therefore, this paper establishes the model through ANSYS Workbench, the dimensions and loads of sign pole are shown in Figure 8.

The panel, column and beam of traffic sign poles are the main areas subject to wind load. The external load will change the natural frequency of the structure^45,46^,so it is necessary to consider the load on the structure when establishing the model. Gravity G is 9.81 $m/s^2$, and wind load is:

1 $$\begin{aligned} F_{wb}=\gamma _0\ \gamma _q\ [\frac{1}{2} \ \rho CV^2\ \sum _{(i=1)}^{n}(W_bi\ H_bi\ )\ ]/1000 \end{aligned}$$

where $$F_{wb} $$is wind load on the sign pole, KN; $$\gamma _0 $$is structure importance coefficient, equal to 1.0; $$\gamma _q $$ is variable load (mainly wind load) component coefficient, equal to 1.4; $$\rho $$is air density, equal to $$1.2258N\bullet S^2\bullet m^{(-4)}; $$ C is coefficient of wind; V is wind speed; n is number of structures;$$ W_{bi} $$is the windward width of the structure, m;$$H_{bi} $$ is the windward height of the structure, m. Workbench uses tetrahedral grids to mesh the model, which has the advantage of being fast, automatically generated, suitable for complex geometries, and easy to refine automatically using curvature and approximate size functions in key areas. In order to ensure the accuracy of the calculation results and reduce the calculation difficulty, the grid size is set as 0.02m, and a total of 522425 nodes and 261290 elements are generated. The important structures of the traffic sign poles are made of Q235 Therefore, the model is also made of this material. The specific parameters are shown in Table 2.

**Table 2:** Performance parameters of Q235.

**Description**	**Value**
Density (kg/m3)	7850
Young’s modulus (Gpa)	200
Poisson’s ratio	0.3
Bulk modulus (Gpa)	166.67
Shear modulus (Gpa)	76.9
Coefficient of thermal expansion	$1.2\times 10^{-5}$

Modal analysis in ANSYS is the solution of the eigenvalues and eigenvectors of the differential equations of a mechanical system whose undamped free vibration should follow the dynamic equilibrium equation^47^:

2 $$\begin{aligned} M\ddot{x}+C{\dot{x}}+kx=f(t) \end{aligned}$$

where M is mass matrix; C is damping matrix; k is stiffness matrix; x is displacement vector; ${\dot{x}}$ is velocity vector; $\ddot{x}$ is acceleration column vector; f(t) is force vector. The motion equation of undamped modal analysis is:

3 $$\begin{aligned} M\ddot{x}+Kx=0 \end{aligned}$$

The free vibration of the structure makes the displacement satisfy the sine function, so

4 $$\begin{aligned} x=x_0sin\omega t \end{aligned}$$

Eq. 4is the solution to Eq. 3, and the natural frequency is f=$\frac{w_i}{2\pi }$, and $\left\{ x\right\} _i$is vibration mode.

5 $$\begin{aligned} \ddot{x}=-\omega ^2x_0sin\omega t \end{aligned}$$

Taking Eq. 5into Eq. 4 gives:

6 $$\begin{aligned} \left( K-\omega ^2M\right) x_0=0 \end{aligned}$$

When the structure vibrates, the amplitude of each node is not all zero, that is:

7 $$\begin{aligned} \left| K-\omega ^2M\right| =0 \end{aligned}$$

From this, the natural frequency of the structure can be solved. The column is an important structure of the traffic sign pole, so its natural frequencies are studied in the paper. In order to prevent the influence of the calculation results, the panel motion was constrained, and 1st to 5th natural frequencies were calculated. As can be seen from Table 3, the 1st and 5th natural frequencies have a large error, but the error of the 2nd natural frequency is small. Therefore, the 2nd natural frequency obtained from Xiaomi 11 is taken as the research object.

**Table 3:** Natural frequency comparison.

**Mode**	**Xiaomi 11 (Hz)**	**HONOR 9 (Hz)**	**HUAWEI (Hz)**	**Simulation (Hz)**
1	6.6	3.2	3.2	9.80
2	23.3	10.2	9.8	25.56
3	-	-	-	36.17
4	-	-	-	66.49
5	79.6	139.7	118.0	83.15

**Figure 9:**
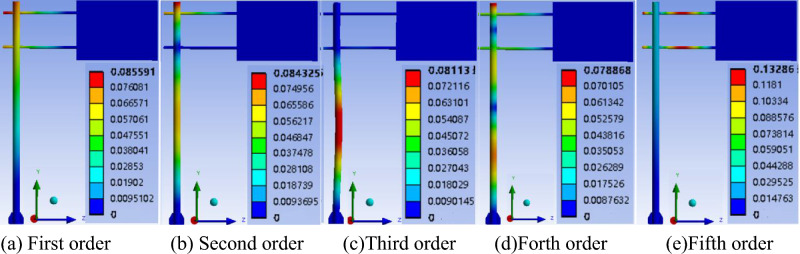
The 1st to 5th natural vibration deformation nephogram.

Modal analysis of ANSYS is used to analyze traffic sign pole. For the reason that low order natural frequencies are important parameters for structures, the 1st to 5th order natural frequencies are studied. According to Figure 9, the 1st, 2nd and 4th order self-vibration of the traffic sign pole are mainly in the form of bending of the column in the X direction. The 3rd order of self-vibration is the bending of the column in the Z direction. The 5th order self-oscillation form is the bending and twisting of the beams in the Y direction. It can be seen that the column is the main structure of the sign pole to occur self-vibration. This may be because the column is the load-bearing part of the traffic sign pole, which makes it more prone to self-vibration.

### Deterioration analysis of sign poles

**Figure 10:**
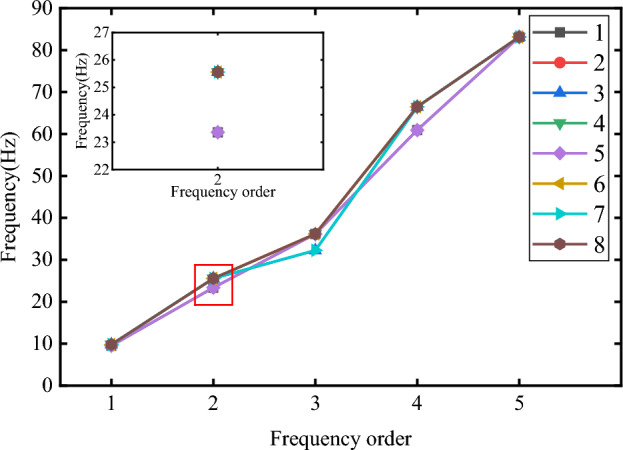
Change in natural frequency of the pole due to different loose bolts.

Fatigue tests on signal structures conducted by Fisher et al^48^ found that all of the fatigue failures occurred at the toe of the fillet weld on the mast arm or pole and that most specimens had significant cracks at both locations. Therefore, it is meaningful to note the connection of base and traffic sign poles. The base of the traffic sign pole is fixed by flanges and bolts, the size and distribution are shown in Figure 8. Under the action of long-term wind load, gaps are easily created between the bolts and flanges, and the loosening of the base connection will lead to changes in natural frequency. In order to study the influence of the loosening of the bolts at different locations on the structure, this paper re-leased the restraint of bolts 1 to 8 in turn, the results are shown in Figure 10. As shown in Figure 10, the 2nd natural frequency varies more than the 1st and 5th natural frequencies. This indicates that the 2nd natural frequency will be more affected by the connection loosening and can be used to quantify the degree of base connection looseness. It was also found that the loosening of bolts 1 and 5 caused a large change in the natural frequency. This is because the bolts perpendicular to the panel direction will be subjected to higher wind loads. Thus, their loosening will lead to an increase in the instability of the structure. The result is the same as Kaczinski et al^49^ and McDonald et al^50^ . They also found that cantilevered sign and signal support structures are susceptible to large displacement amplitudes resulting from galloping.

### Prediction of deterioration of sign poles

**Figure 11:**
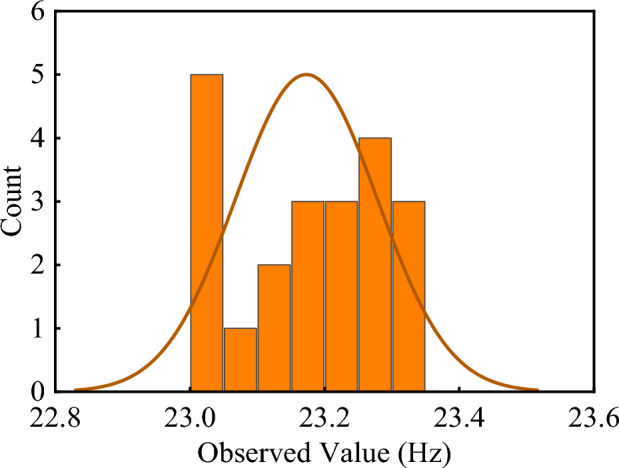
Normal distribution of 2nd natural frequency.

**Figure 12:**
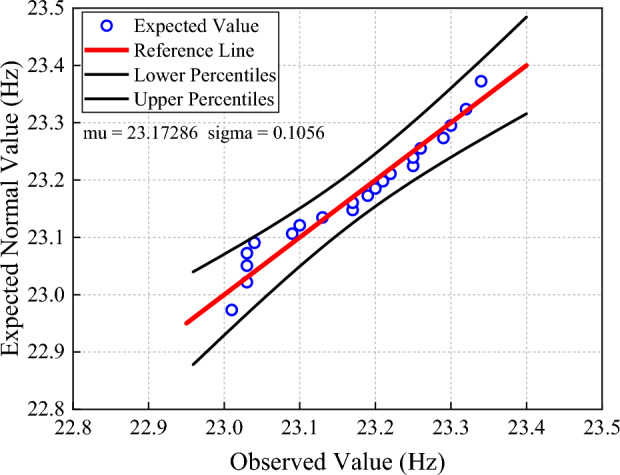
Normal Q-Q Plot of 2nd natural frequency.

To test the stability of the natural frequencies of the sign pole, the 2nd natural frequency was collected three times a day for a week, and the samples were subjected to a Shapiro-Wilk test. Shapiro Wilk test is a normal analysis method, mainly applicable to small sample analysis^51–54^. The result showed that the sample significance is 0.181, which is far greater than 0.05. It means the samples meet the normal distribution as shown in Figure 11. Then the samples were subjected to a Quantile-Quantile Plot analysis with a confidence level of 95% as shown in Figure 12. Q-Q plot is a probability plot that compares two probability distributions graphically. If the two distributions are linearly correlated, the points tend to fall on a straight line on the Q-Q plot^55–58^. The vertical coordinate is the predicted value that fits the normal distribution and the horizontal coordinate is the observed value. Q-Q plot shows that the samples conform to the normal distribution and fall within the upper and lower percentile, indicating that the 2nd natural frequency of the sign pole is stable.

**Figure 13:**
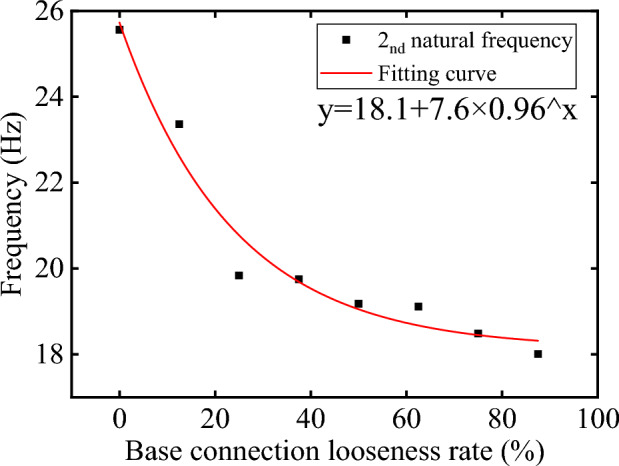
The 2nd natural frequency fitting.

The deterioration of traffic sign poles is mainly due to looseness of base connection. The looseness of the base connection will lead to a change in the natural frequency, so the deterioration degree of the sign pole can be obtained by studying the change in the natural frequency. It can be seen from the above that the 2nd natural frequency can be accurately obtained by the mobile phone, and 2nd natural frequency is more sensitive to the looseness of the base connection, so it is taken as the standard to measure the deterioration. This paper simulates the condition of base connection looseness by removing 1 to 7 bolts, which is equivalent to removing 12.5% to 87.5% of connection constraints. According to the change of the 2nd natural frequency, the formula fitting shows that $R^2$ is 0.96, indicating that the curve fitting is good. Figure 13 shows that when one or two bolts are removed, the natural frequency varies significantly, up to roughly 5.7Hz, but when more bolts are removed, the natural frequency slowly declines.

Therefore, when checking the health of traffic sign poles, the 2nd natural frequency should be measured and compared with its theoretical value. The sign pole is considered healthy if the measured value is within the error range of the normal distribution. If the measured value deviates greatly from the theoretical value, it is considered that the sign pole has deteriorated. The deterioration degree can be obtained through the fitting formula and can judge whether the sign pole needs to be replaced or repaired.

## Conclusions

This paper tests the performance differences between the acceleration sensors of three mobile phones and traditional acceleration sensor. Through shaking table tests and on-site and numerical simulation tests on traffic sign poles, it was found that the acceleration sensor of mobile phones has excellent performance. Based on experiments, a convenient and effective method to monitor the health of traffic sign posts by using mobile phones is proposed. The conclusions and discussion are as follows.

(1) The LSM6DSO sensor of the Xiaomi 11 offers high performance. Although the measured acceleration amplitude is slightly smaller than the actual value obtained by traditional acceleration sensor, the waveform is highly consistent. The Pearson correlation coefficient of curves is 0.81, which shows a very strong correlation. Therefore, the Xiaomi 11 can be used as a conventional acceleration sensor under the similar sampling frequency.

(2) Xiaomi 11 can obtain the 1st, 2nd and 5th natural frequencies of traffic sign poles. However, the 1st and 5th natural frequencies have a large error, while the 2nd order is close to the self-frequency obtained from traditional acceleration sensor and simulation test. The samples of 2nd natural frequency are stable and conform to the normal distribution.

(3) The bolts perpendicular to the panel direction have a more significant impact on the natural frequency of the structure. This greater impact is due to the fact that they bear a higher stress ac-cording to the wind loads, and their looseness will lead to structure instability. Consequently, throughout the inspection, focus should be given to these bolts.

(4) The looseness of the base connection has a large effect on the 2nd natural frequency. Especially when one or two bolts are removed, the 2nd natural frequency changes significantly, which is up to 5.7Hz. However, with the further release of the constraint, the change of the 2nd natural frequency tends to be gentle. Therefore, it is taken as a measure of base connection looseness. The formula fits well and can be used to predict the deterioration of a traffic sign pole.

## Discussion

This paper tested the performance difference between mobile phone acceleration sensors and traditional acceleration sensor. It was found that there was little difference of the acceleration curve. However, due to dynamic fluctuations in the sampling frequency of mobile phone acceleration sensors, the natural frequency is significant different from traditional acceleration sensor, except for the 2nd self- frequency. The change of the 2nd self- frequency of the traffic sign pole is used to determine its health degree, because the 2nd self- frequency is more sensitive to the loosening of the base. The further study should be carried out to investigate the influence of other kinds of damage on the structure self-frequencies, which can be used to comprehensively judge the health degree.

## Data Availability

The data presented in this study are available on request from the corresponding author.
